# Biologically Inspired Collagen/Apatite Composite Biomaterials for Potential Use in Bone Tissue Regeneration—A Review

**DOI:** 10.3390/ma13071748

**Published:** 2020-04-09

**Authors:** Barbara Kołodziejska, Agnieszka Kaflak, Joanna Kolmas

**Affiliations:** Chair of Analytical Chemistry and Biomaterials, Department of Analytical Chemistry, Medical University of Warsaw, ul. Banacha 1, 02-097 Warsaw, Poland; barbara.kolodziejska@wum.edu.pl (B.K.); agnieszka.kaflak@wum.edu.pl (A.K.)

**Keywords:** collagen, hydroxyapatite, biomimetic material, scaffold, bone regeneration, biocomposite

## Abstract

Type I collagen and nanocrystalline-substituted hydroxyapatite are the major components of a natural composite—bone tissue. Both of these materials also play a significant role in orthopedic surgery and implantology; however, their separate uses are limited; apatite is quite fragile, while collagen’s mechanical strength is very poor. Therefore, in biomaterial engineering, a combination of collagen and hydroxyapatite is used, which provides good mechanical properties with high biocompatibility and osteoinduction. In addition, the porous structure of the composites enables their use not only as bone defect fillers, but also as a drug release system providing controlled release of drugs directly to the bone. This feature makes biomimetic collagen–apatite composites a subject of research in many scientific centers. The review focuses on summarizing studies on biological activity, tested in vitro and *in vivo*.

## 1. Introduction

Bone grafting, which is performed to regenerate bone tissue and to treat bone defects with various origins, remains one of the most commonly performed surgical procedures. Every year, around two million bone grafts are carried out worldwide, which shows the great need to develop this branch of medicine [1,2]. The start of the development of bone implantology dates back to 1913, when an attempt was made to implant a fragment of a cat bone and a human bone into a dog’s body. The overgrowth of implants with newly created bone tissue was considered a great success; therefore, research on xenografts, allografts and autografts intensified. Xenografts involve transplanting an organ, tissue or cells to an individual of another species. An allograft is a bone or tissue that is transplanted from one person to another. [3]. Today, due to the absence of autoimmune reactions, high osteoinduction (the ability to induce the osteogenesis process) and osteoconductivity (the bone growth), the “gold standard” is the autologous graft (transplant comprised of an individual’s own tissue). However, it should be emphasized that its use is highly limited [3,4,5,6,7]. Allografts and xenografts are not only associated with the risk of autoimmune reaction and consequently rejection of the implant, they are also unable to meet the demands of the treatment of bone tissue defects. Artificial bone substitutes have become a solution to these restrictions. The great advantages of these materials are their unlimited production and control of their physicochemical and biological properties [4].

A variety of implant materials are used in bone restorative surgery, both biodegradable and non-degradable. These materials can be a permanent filling or a tissue connector. The primary requirements for biomaterials used as implants are: non-toxicity, durability, biocompatibility (blood compatibility), resistance to platelet and thrombus deposition and being non-irritating to tissue. Moreover, they should be chemically stable and bio-inert. The most commonly used materials are metallic, ceramic and polymer materials [8,9]. Unfortunately, none of these materials meet all the requirements for implant biomaterials. Metals are often too stiff relative to bone tissue and unfavorably corrode in the body. Ceramic materials, despite their high biocompatibility and bioactivity, are characterized by poor strength and high fragility, and cannot be used in places subject to high stress. Polymers are often characterized by over-flexibility and low strength in relation to mineralized bone tissue. To maintain appropriate mechanical properties, a variety of composite materials are created, usually containing a polymer phase (providing flexibility) and a ceramic phase (providing hardness and strength) [9,10].

It is worth emphasizing that in creating synthetic bone substitutes, the key requirements for a good scaffold are the biocompatibility of the material, its osteoconductivity and its osteoinduction [11]. The way to achieve the appropriate biological, physicochemical and mechanical parameters is to create biomimetic materials, inspired by the chemical composition and the micro and ultra-structure of bone tissue [11,12]. Collagen–hydroxyapatite (HA/Col) composites are this type of material. Type I collagen and calcium phosphate in the form of apatite are the main components of bone and can be used in the production of bone tissue replacements. Research shows that such biomaterials have good biological and mechanical properties [13,14]. HA/Col composites can serve not only as a scaffold for newly formed bone, but also as a carrier of drugs, delivering them directly to the bone [15]. What is more, the development of 3D printing techniques makes it possible to create implants for the patient’s individual needs (printing scaffolds with a specific shape and porosity) [16,17].

In the present paper the state of knowledge about HA/Col composites was studied. The work is both a review of the basic methods of obtaining these biomaterials and the state of knowledge about biological properties (in vitro and *in vivo*). This review focuses on composites containing hydroxyapatite and collagen (bone tissue components). Further work is planned to summarize the current literature on HA/Col composites with the addition of other synthetic components.

## 2. Bone Tissue

**Figure 1:**
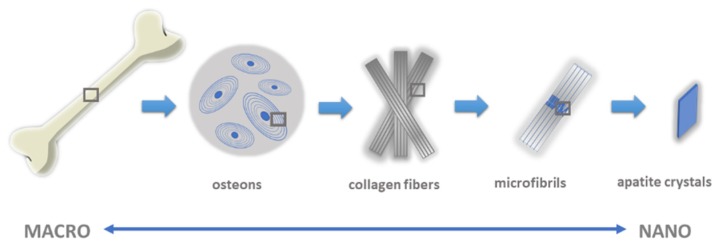
The multi-scale structure of natural bone.

Bone tissue is a diverse form of connective tissue with high metabolic activity, heterogeneous and dynamic structure and high mechanical strength [18,19]. It is made of extracellular substances and bone cells: osteoblasts, osteoclasts, osteocytes and osteogenic cells. Bone tissue co-creates the locomotor system, protects internal organs and bone marrow and stores mineral salts (99% calcium, 88% phosphorus, 50% magnesium and 35% sodium are located in bone tissue) [19]. Adaptation of bone structure to perform such important functions includes a number of organizational levels. These include: the molecular structure and distribution of crystals and organic components (nanoscale); the structure of bone plates; the structure and arrangement of spongy bone tissue and osteons of compact bone tissue; and macroscopic structure (macroscale) (Figure 1) [20,21].

The bone has a hierarchical structure: from the level of the whole tissue, i.e., the occurrence of various types of long and short bones, flat or tubular, to the level of the tissues which are arranged in cortical and spongy structures, through to the microscopic level (images of cells, matrices and minerals) to the level of nanometers from single bone apatite crystals and collagen fibers [20,22].

The osteon is the main structural and functional unit of compact bone. Its structure is made of 6–15 cylindrical bone plates arranged concentrically around the central channel (Haversian canal). The interior of the canal is filled with individual osteogenic cells, osteoblasts and osteoclasts. The diameter of the channels is 20–100 μm. Bone plates are made of parallel fibers, mainly built with collagen type I mineralized with nanocrystalline multisubstituted carbonate hydroxyapatite (so-called bone apatite). Bone tissue also includes other non-collagen proteins and water. In general, apatite ensures bone hardness, while the organic fraction forms the scaffold for the biomineral and regulates the biomineralization process [21].

Bone apatite, i.e., biological apatite, is a mineral with a specific chemical composition that determines the biological, physicochemical and mechanical properties of the entire tissue. It is a nanocrystalline carbonate hydroxyapatite, additionally containing a variety of different ions (e.g., Mg^2+^, K^+^, Na^+^, Mn^2+^, HPO_4_^2−^ and SiO_4_^4−^) [23,24,25,26]. The organic fraction of bone tissue is mainly made up of type I collagen [27]. The latter consists of three polypeptide chains entwined to form a triple helix. This structure is a so-called superhelix, with the occurrence of characteristic fragments containing repeating sequences: Gly–Pro–Hyp. Five triple helices assemble with each other, creating a microfibril. The microfibrils then organize into fibrils, forming compact fibers with diameters of about 100–200 nm. Collagen fibers also crosslink via lysine residues. The ordered fibrillar system is stabilized by other non-collagen proteins [27]. In the free spaces of collagen fibers, apatite crystals with a width of 15–30 nm, a length of 30–50 nm and a thickness of 2–10 nm are settled. They are composed of a crystalline core and a hydrated surface layer. The hydrated surface layer is about 1–2 nm thick and contains various ions. Recent studies show that the components of the hydrated surface layer are responsible for apatite reactivity, adsorption properties and the process of crystal maturation and growth. It is noteworthy that this is also the border region between crystalline apatite and the organic matrix of bones [28,29,30,31]. The small sizes of the crystals and the presence of such ions as CO_3_^2−^, Na^+^ and Mg^2+^ (in amounts of approximately 4–6%, 0.9% and 0.5%, respectively) means that particles of natural hydroxyapatite are easily absorbed [32].

Among non-collagen proteins of extracellular bone tissue (constituting about 5% of the organic matrix and synthesized by osteoblasts or other cells, or reaching bone tissue with blood), we distinguish proteoglycans (mainly chondroitin sulphate), which can affect the formation, thickness and orientation of collagen fibers, and are also for the binding of hydroxyapatite, osteonectin and osteocalcin, and fixing proteins such as fibronectin, osteopontin and bone sialoprotein (BSP) [18,19].

In addition to the organic matrix (approximately 20–30% by weight) and the mineral fraction (representing 60–70% of bone mass and consisting primarily of nanocrystalline apatite), the third equally important component of bone tissue is water. It constitutes about 10% of bone mass. It facilitates fluid transport, contributes to elastic properties and plays a key role in the mineralization process. Water is on the surface of mineral crystals, inside the crystals and between collagen fibers. Most of the water is located in the pore spaces (so-called associated water), whose content decreases with bone age. Apart from water associated with bone tissue, there is also bound water, located in the organic matrix; i.e., in type I collagen fibers and in bone mineral.

It is also worth noting that bone tissue is a dynamic structure whose composition is not constant. The content of water in bone tissue is also variable. This can affect the properties of collagen (its elasticity); the coherence of the connections of bone composite components; and the ion exchange and balance between bone formation and bone resorption. This is why the formation of new biomimetic HA/Col biocomposites which imitate biological bone tissue is so important [23,27].

## 3. Synthesis of Collagen–Apatite Composites

When producing HA/Col composites, one should aim to produce a material that closely resembles the chemical composition, micro and macro-structure and porosity of the natural composite, i.e., bone tissue. Thanks to these biomimetic properties, materials with high compatibility as well as osteoinduction and ocostoconductivity can be obtained [4,33]. There are several methods for obtaining HA/Col composites [34].

Among them, the most basic method is the simple mixing of previously obtained apatite powder with a collagen solution. Apatite can be synthesized in many ways. There are many reviews on the preparation and properties of hydroxyapatite and hydroxyapatite enriched with various ions [35,36,37,38]. Usually, wet methods are used, which involve the precipitation of calcium phosphate from appropriate reagents (e.g., calcium nitrate and ammonium phosphate, as sources of calcium and phosphorus, respectively) added in the appropriate ratio (Ca/P molar ratio = 1.67) and at the appropriate pH (usually pH > 8). The precipitate, after an appropriate aging time, is subjected to filtration, drying and heating at a suitable temperature. It is worth noting that the concentrations of reagents, temperature, pH and aging time influence the size of the crystals obtained and their morphology [35,36].

Type I collagen is often used to make composite materials (this is the organic matrix of bone tissue). Collagen can be obtained from pig skin, bovine or horse tendons, rat tails, etc. [39]. In some works, atelo-collagen was used, which was obtained after the enzymatic treatment of collagen and removal of telopeptides (to minimize antigenecity) [40,41]. Atelo-collagen is often more soluble and forms a collagen solution, whereas type I collagen forms a suspension. It is also worth mentioning that collagen used as an individual material does not have osteoinductive properties, but acquires them in combination with calcium phosphate (apatite) [3,4]. After mixing the gel/collagen solution with apatite powder, a suspension is formed, which is then subjected to drying at a critical point, or lyophilization. Of course, many authors have applied modifications of this method [42,43,44,45,46,47,48,49,50,51,52,53,54,55].

An interesting comparison of two methods for the preparation of HA/Col composites was presented by Cuniffe et al. [42]. In the first method, nanohydroxyapatite particles were added to the collagen suspension (slurry-suspension method) and then lyophilized, while in the second method, the lyophilized collagen in the form of a porous scaffold was soaked in nHA suspension and then lyophilized (immersion method). In both cases, composite scaffolds with highly porous, interconnected structures were obtained. It was found that the suspension method was more repeatable and easier to perform. In a paper by Uskoković et al. [43], hydroxyapatite was obtained by the reaction of ammonium phosphate and calcium nitrate and then calcinated at 1100 °C during 6 h. The hydroxyapatite was mixed with type II collagen in a mortar and then pressed into pellets at room temperature and 60 °C. The resultant composite material was subjected to physicochemical analysis. SEM analysis showed that the material pressed at a higher temperature is characterized by more intimate contact between collagen type I and apatite phases.

**Figure 2:**
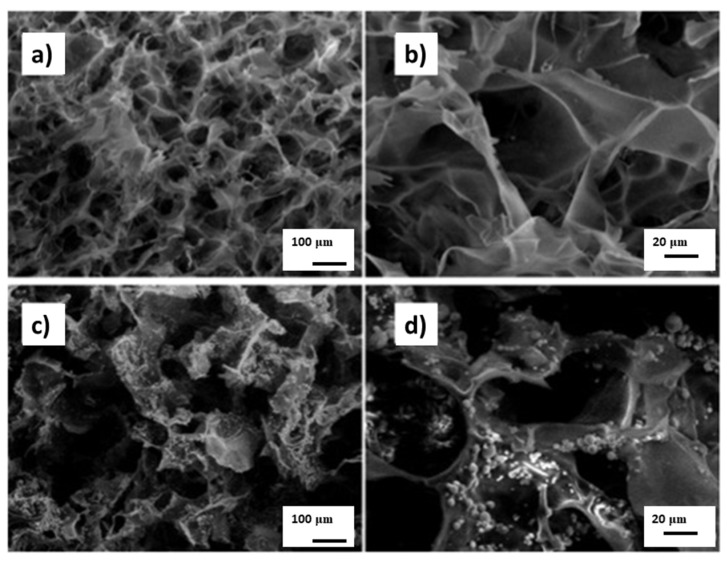
SEM of scaffolds. (**a**,**b**) Pure collagen. (**c**,**d**) Col/mHA (collagen/hydroxyapatite-microsphere). Scale bars: 100 μm (**a**,**c**), 20 μm (**b**,**d**). Reprinted from [44] with permission from Elsevier.

In a paper by Cholas et al. [44], hydroxyapatite microspheres obtained by spray drying were used to produce the HA/Col hybrid composite. The paper suggests the possibility of using such a composite as a carrier for a drug substance that would be placed in the mesoporous structure of the microspheric HA (Figure 2).

Since 3D porous materials are characterized by a spongy structure, an interesting solution was proposed by Teng et al. [45]. Type I collagen dissolved in 1,1,1,3,3,3-hexafluoro-2-propanol (HFP) was added to the aqueous suspension of hydroxyapatite. Titanium discs after previous cleaning were coated with a homogeneous mass of a composite in a spinner, followed by drying the coated Ti substrates in a desiccator under vacuum overnight. Subsequently, the coatings were chemically cross-linked in two solutions: N-(3-dimethylaminopropyl)-N0-ethylcarbodiimide (EDC) hydrochloride and N-hydroxysuccinimide (NHS). The composite coatings obtained were characterized by high homogeneity, while the sample containing 20% hydroxyapatite turned out to be the most hydrophilic.

Tampieri et al. obtained (HA/Col) composites by two methods. In the first, a collagen suspension was mixed with hydroxyapatite previously obtained by precipitation from Ca(OH)_2_ solution with H_3_PO_4_ solution [46]. In the second method, the precipitation of hydroxyapatite from the reagents used in method I was carried out in the presence of collagen. From the results, it can be concluded that the method based on the direct precipitation of apatite in collagen solution is best. The resultant material has much greater similarity to bone tissue, and the collagen fibers are in close connection with apatite crystals. The material obtained by simply mixing the components is characterized by properties similar to those of collagen. In addition, it is worth noting that according to the SEM results, the composite obtained by the standard method (simple mixing hydroxyapatite with collagen) has a less homogeneous structure with apatite crystals distributed unevenly on the surface of collagen fibers.

The method with the precipitation of hydroxyapatite in the presence of collagen has been repeated by many researchers [30,56,57,58,59,60].

**Figure 3:**
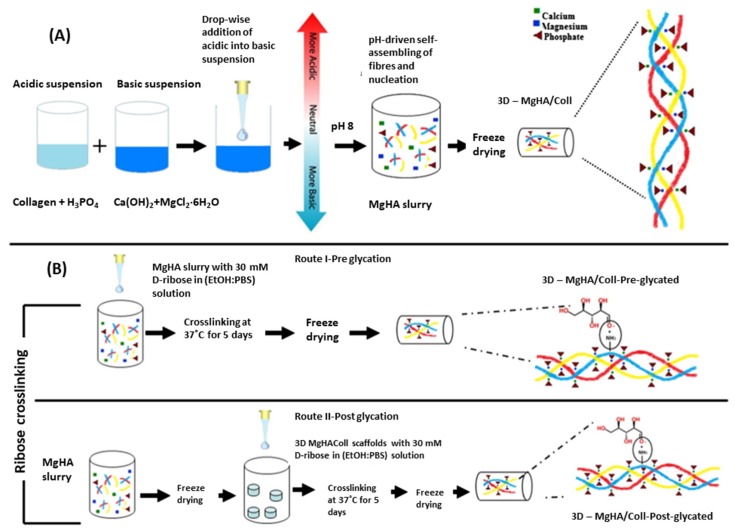
Detailed schematic illustration of the MgHA/Coll (type I collagen matrix with magnesium-doped-hydroxyapatite nanophase) hybrid scaffold’s development: (**A**) pH-driven, bioinspired biomineralization process; (**B**) MgHA/Coll crosslinking with ribose scaffolds in pre and post-glycation processes. Reprinted from [57] with permission from Elsevier.

An interesting modification was developed by Yunoki et al. [56]. Synthesized under standard conditions (phosphoric acid and Ca(OH)_2_ as reagents for HA and atelo-collagen type I mixed together at pH 8–9 and temperature around 40 °C), self-organized nanocomposite HA/Col (after initial lyophilization) was placed in distilled water or in PBS buffer (standard PBS, pH = 7,4) and then re-frozen and placed under vacuum at 140 °C. In the presence of PBS, more effective crosslinking of collagen fibers occurred and porous composites with very good mechanical properties were obtained. According to Krishnakumar et al. [57], ribose can be used to successfully crosslink the HA/Col composite structure (Figure 3). In this work, MgCl_2_•6H_2_O was used as a source of magnesium ions introduced into the structure of synthesized apatite in order to obtain a highly compatible material with bone tissue. In research by Calabrese et al. [58], magnesium ions were also used in the production of the composite, but crosslinking was carried out using bis-epoxy (1,4-butanediol diglycidyl ether, BDDGE). Glutaraldehyde was another reagent that was used to crosslink the obtained hybrid composites [30,49].

A completely different approach to creating a composite 3D structure was presented by Zhou et al. [59]. To improve the mechanical properties of the composite, a porous ceramic matrix of hydroxyapatite and beta-tricalcium phosphate (beta-TCP) was first formed. Then the ceramic 3D material was soaked in a collagen and SBF suspension at pH 4–6 and then completely immersed in a collagen solution and closed under high pressure. A vacuum infusion was carried out at a pressure of 10 Pa and held for 2 h to allow complete saturation of the samples. The scaffolds were freeze-dried and then crosslinked with glutaraldehyde.

## 4. Biological Properties of Apatite–Collagen Composites

Of course, a key aspect of research on HA/Col composites is obtaining information on their biological properties and the potential uses of the resulting biomaterials. Recently, there have been more studies on this topic. Preclinical and clinical studies using various in vitro and in vivo models provide important information on bone tissue regenerative properties; bioresorbability; and the impacts of the elasticity and porosity of such bone substitutes in their micro and macro-environments.

### 4.1. Osteoconductivity, Osseointegration, Bioactivity and Biocompatibility

There are many studies confirming the ability of HA/Col composites to stimulate the formation of new bone tissue. In all biological tests for the production of HA/Col composites, type I collagen (atelo-collagen extracted from porcine dermis or bovine tendon) was used. In one study, Fukui et al. implanted composites consisting of nano-HA and collagen into the mandibles of rabbits [61]. Collagen sponge and collagen sponge/calcined hydroxyapatite composite were used as controls. Calcined HA was prepared by heating nano-HA at 900 °C for one hour. Histological examination showed a greater amount of newly formed bone tissue in the nano-HA/Col composite environment than in controls, and faster implant replacement with host bone tissue, which also confirms the high bioresorbability of the material.

**Figure 4:**
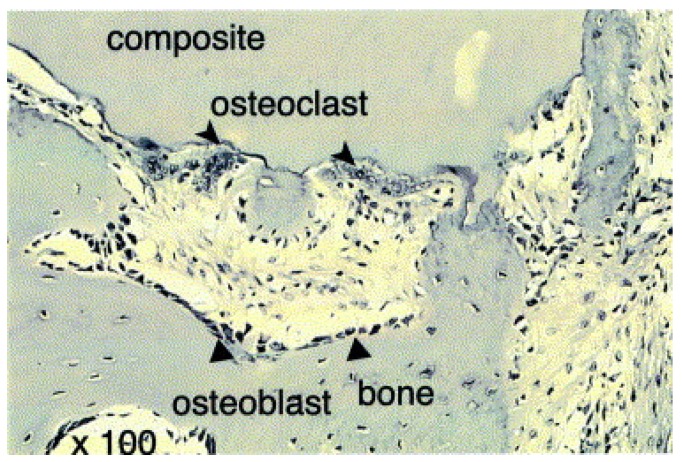
HE-stained histological section (×100) of HAp/Col composite implanted into a beagle’s tibia for 12 weeks. Triangles indicate elongated cells, and arrowheads multinucleated giant cells. Reprinted from [40] with permission from Elsevier.

The purpose of the research by Kikuchi et al. [40], was to synthesize a composite material (based on hydroxyapatite and collagen) as similar as possible to bone tissue and to use it for testing on dog tibia bones. In the synthesis of composites, the optimal pH and temperature were selected to improve the mechanism of collagen fiber organization. The composite was introduced in place of a 20 mm defect. Bone condition was observed using X-ray techniques for 12 weeks. In the place of the composite, the newly formed bone tissue gradually penetrated, and the defect was completely filled after 8 weeks. After 12 weeks, the bone and the composite were removed and the material was observed with a microscope (Figure 4). Two types of cells near the composite were observed— osteoblasts and osteoclasts. The composite was included in the bone remodeling process, which so far has mainly been observed for autografts. Such high bioactivity of the material may be the result of high similarity to bone tissue. The composite can be recognized as bone by the surrounding cells. Nishikawa conducted research on a similar group (dog tibia bones) [62]. The stimulating effect of such composites on the synthesis of new bone tissue has been proven. It is concluded from the results of the study that HA/Col may be a source of calcium ions that are incorporated into the newly formed bone tissue.

**Figure 5:**
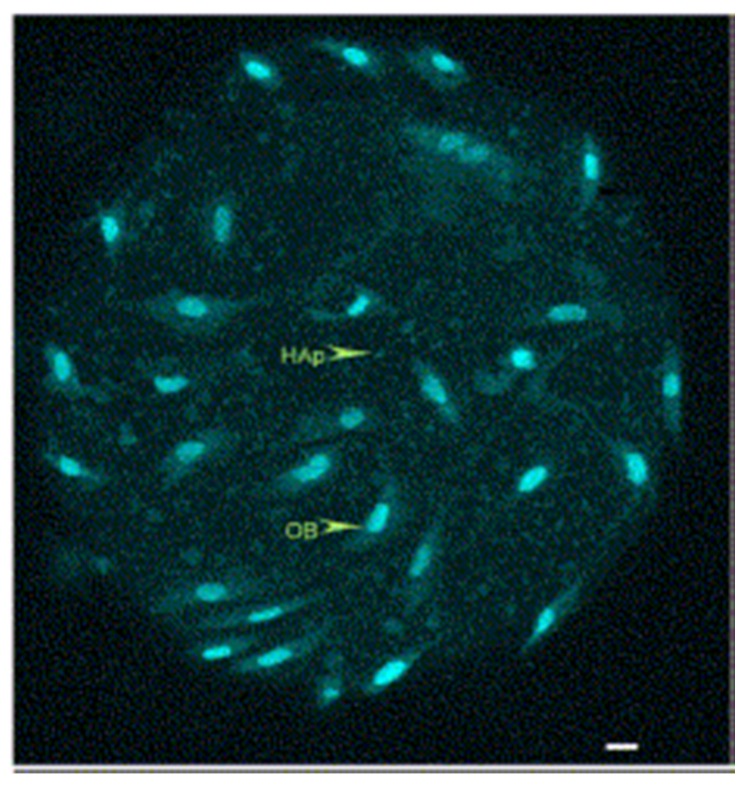
Confocal microscopy of osteoblast cells cultured on microsphere staining with DNA dye YOYO-1 (HAp: hydroxyapatite, OB: osteoblast; 4 days after seeding). Reprinted from [47] with permission from Elsevier.

An important property when describing the biological functions of HA/Col composites, is their ability to support migration of cells from surrounding tissues. Yoshida et al. studied the adhesion, proliferation and osteogenic response of MG63 cells using 3D sponges, high porosity HA/Col and as a control, collagen sponge [63]. The cells with sponge were examined by histology, total DNA content and gene expression. The results suggest that materials based on hydroxyapatite and collagen have good osteogenic properties and can successfully serve as scaffolds in bone tissue reconstruction. The total DNA content in the HA/Col sponge was 1.8 times higher than in the control sample, and the osteogenic cells showed good and even adhesion over the entire surface of the sponge. HA/Col composites create a space that facilitates precursor cell migration, proliferation and differentiation. The results were confirmed by another study using a HA/Col membrane [64]. Similarly, bone marrow cells were cultured together with osteoblasts on the HA/Col composite and differentiation to osteoclasts was observed without the addition of other factors. That distinguishes this material from pure HA or TCP [65]. Wu et al. prepared the HA/Col composite in the form of microspheres, and with this model also proved that osteoblasts are capable of proliferation, differentiation and mineralization in the matrix of microspheres (Figure 5) [47].

Calabrese et al. implanted HA/Col composites in mice. Hydroxyapatite was additionally enriched with magnesium ions [58]. The results indicate the ability of this material to recruit host cells and promote ectopic bone growth *in vivo*. Correct angiogenesis was also confirmed by FMT analysis. The authors emphasize that the materials are characterized by a high degree of safety, due to the lack of the addition of growth factors or cells subjected to in vitro manipulation, and they can be a safe and promising solution in the treatment of bone diseases [66].

There is a special, commercially available bone substitute biomaterial (Biostat) with the composition: hydroxyapatite, collagen and chondroitin sulphate. In a clinical trial, two groups were followed: group A—filling the defect with Biostat; group B—no defect fill [67]. After 4–6 months, the group treated with Biostat implants showed a higher percentage of new bone tissue coverage (67%) compared to group B (34%). Another study confirms that Biostat material affects bone tissue reconstruction [68].

Materials with the best biocompatibility are still being sought. Certainly, the greater the similarity to physiological bone tissue, the more likely it is that tissue compatibility will be achieved. It is presumed that the carbonate content of apatite affects the formation of new bone tissue by affecting the solubility and crystallinity of apatite [69]. Biological apatite contains about 4–6 wt% of carbonates [24]. Matssura et al. proved that among the HA/Col composites with different carbonate contents, the one with the content of 4.8% could be distinguished from the others [48]. Composites containing this apatite had the greatest ability to form new bone tissue after implantation in the femurs of rabbits. This was observed on the X-rays and examined using histology. It was also concluded that bone metabolism is strongly associated with the physicochemical properties of apatites, especially with the carbonate content.

Mazzoni et al. assessed the biocompatibility, osteoconductive and osteoinductive properties of HA (Pro Osteon 200) and collagen (Avitene) composites using a cell model—mesenchymal stem cells (hMSC) [70]. Expression of osteogenic genes was analyzed in cells located on the composite. The results showed that such biomaterial has the ability to induce osteogenic differentiation of hMSC, because it induces osteogenic genes and increases matrix mineralization without toxic effects. Other studies also conducted on the cell line have confirmed that the HA/Col composite has osteoinductive properties and is a good tool to accelerate the migration, proliferation and differentiation of bone tissue cells [71]. The above material was also used in maxillofacial surgery as a kind of scaffolding for the zygomatic bone. The high biocompatibility and the osteoconductive properties of the composite have been confirmed and the low number of postoperative infections has been noted [72].

Initial tests have been carried out to check the bone density change after using various defect replacement materials. One study used computer tomography (CT) to evaluate extraction site dimensions and density changes after a tooth extraction. Different graft materials were tested [73]. Patients were divided into three groups. The first group was treated with a demineralized bone matrix with the addition of collagen membrane and the second with hydroxyapatite with the addition of collagen membrane; in the third group the extraction site remained empty. A CT scan was performed 10 and 120 days after surgery. It was shown that the use of HA/Col gives the highest bone density in CT compared to the use of demineralized bone matrix and group III. However, no changes in vertical socket dimension were observed. Still, the authors of the study say that HA and collagen-based material could be recommended to improve bone quality and could prepare the extraction site properly for proper implant placement.

**Figure 6:**
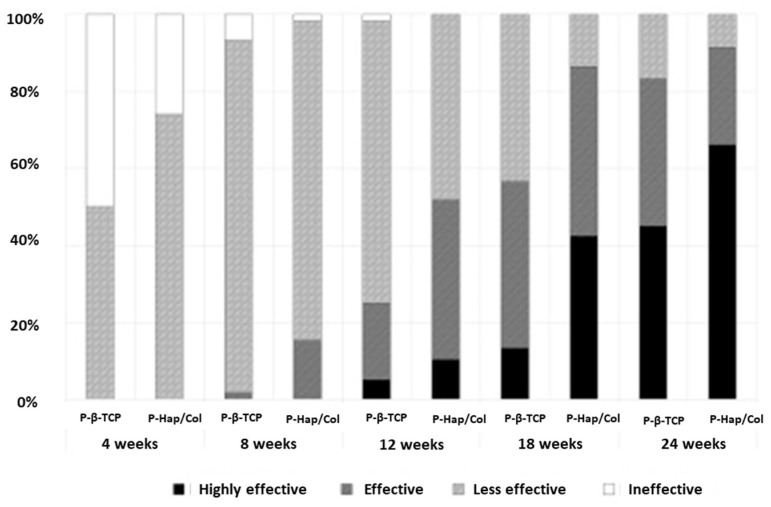
Results of X-ray evaluation. The scores improved over time during the follow-up period in both groups. At each time point, the score in the HAp/Col group was higher than that in the β-TCP group. Reprinted from [74] with permission from Elsevier.

Clinical studies have also compared HA/Col and β-TCP as implants in patients after a history of bone cancer or fractures [74]. The effectiveness of the materials was evaluated by using X-rays to assess bone regeneration. This study proved the superiority of porous HA/Col over β-TCP by presenting the results of bone regeneration and implant resorption (Figure 6). In contrast to β-TCP, the material containing collagen adapted to the shape of the bone defect, leaving no gaps or free spaces, and connected continuously with the bone. There were more side effects for HA/Col than for β-TCP, but they were not serious.

### 4.2. Bioresorbability of Composites

Biodegradation or bioresorbability are very desirable processes when designing implant biomaterials. Due to such properties, the implanted material quickly disappears and is replaced by newly forming host bone tissue. Biodegradation has been studied using composite membrane carbonate apatite–collagen, in vivo and in vitro [75]. With the in vitro method, the membranes were immersed in collagenase solution and the degradation time of the composite was analyzed. The results showed a gradual increase in the concentration of calcium ions, which is related to and dependent on the dissolution of the collagen membrane. The membrane was also implanted in rat bone tissue and histological changes were analyzed. Studies have shown good membrane biocompatibility and the biodegradation time has been reduced due to the presence of carbonate apatite. The biodegradation time can therefore be controlled by the carbonate apatite contained in the membrane.

Another study compared the properties of pure hydroxyapatite with a carbonate apatite–collagen composite [76]. Rabbit tibia bone fragments were removed and appropriate materials were implanted. Composites gradually degraded, and the newly formed bone filled the defect within 6 weeks, while the implanted hydroxyapatite was not replaced by bone tissue. There are also other animal studies that confirm the successful osseointegration of HA/Col materials [61,77].

### 4.3. Porosity and Biological Properties

The porosity of HA/Col composites also has a significant impact on biological properties. This was tested on an animal model by implanting porous scaffolds into the tibias of rabbits. Implants with higher porosity were characterized by faster formation of new bone tissue and its penetration into composite structures. The porous structure ensures better osteoconductivity. It allows better cell migration and facilitates the formation of blood vessels that ensure the proper nutrition of newly formed bone tissue [78,79,80]. Porous composites in contact with water become elastic. This property means that they are easy to use in surgery and adapt to the difficult shapes of cavities. However, it is believed that these materials are less mechanically strong due to their porous structures. In the study using HA/Col and TCP, the above materials were implanted in the tibia bones of rabbits to test their biomechanical properties. It was found that although the HA/Col composite is less mechanically durable than TCP, after implantation in the place of defect it showed greater mechanical strength in a given place than the TCP material [81].

Despite the fact that high porosity reduces its mechanical strength, sponge-like elasticity provides easy “handling” during surgery [82]. After wetting, HA/Col porous composites become elastic, like a sponge, and are easily implanted in bone defects [78,83]. From a surgical point of view, the addition of collagen to hydroxyapatite provides many beneficial features during surgery: ease of fitting to the defect site (blocks can be cut), easy adaptation to the defect morphology, the ability to stick material to the transplant site and the ability to promote clot formation and stabilization thanks to the hemostatic properties of collagen [63,79].

### 4.4. Anticancer Properties

There have been reports of the effect of mineralization of collagen fibrils in bone tissue on the adhesion of cancer cells [84,85,86]. These studies are looking for the cause of bone metastases. One approach was to investigate the effect of extracellular matrix bone on metastasis. A study by Siyoung Choi et al. [87] demonstrated that the physiological mineralization of collagen fibrils reduces tumor cell adhesion with potential functional consequences for skeletal colonization of disseminated cancer cells in the early stages of breast cancer metastasis. Too little of the mineral part of the matrix may be associated with an increased risk of bone metastases. The study compared collagen-mineralized fibers and non-mineralized collagen for regulating the adhesion of metastatic breast cancer cells. Endothelial collagen mineralization can change the response of cancer cells first through integrin-mediated mechanotransduction.

## 5. New Trends

In bone diseases, pharmacotherapy is often used in addition to surgical treatment. It is usually associated with serious side effects, and due to weak bone vascularization, therapeutic drug concentration is not achieved [88]. For this reason, composite materials began to be used as drug delivery systems to achieve greater therapeutic effects. There are studies which describe the introduction of anti-resorptive drugs, anti-cancer drugs, antibiotics, proteins or genes [89,90,91,92,93,94,95]. It is worth mentioning that infections occurring in the implant area are a major problem in orthopedic surgery. These infections can lead to disabling or even life-threatening complications. In this situation, the introduction of antibiotics directly to the place of therapeutic effect seems a good solution. Simultaneously, bone defects are treated and infection is prevented. There are reports in the literature of the use of various materials to deliver these medicinal substances to bone tissue. The porous HA/polymer structure appears to be a suitable matrix for delivering antibiotics directly to the bone [96]. These composites have been found to be highly biocompatible and also prolong the release of the substance. As a result, such a composite was recognized as an effective carrier of antibiotics to control bone tissue infections, while supporting bone regeneration [89,90]. Due to the types of infection found in bone tissue, the most commonly selected and tested antibiotics in combination with the composite are gentamicin and vancomycin [91,94,97].

**Figure 7:**
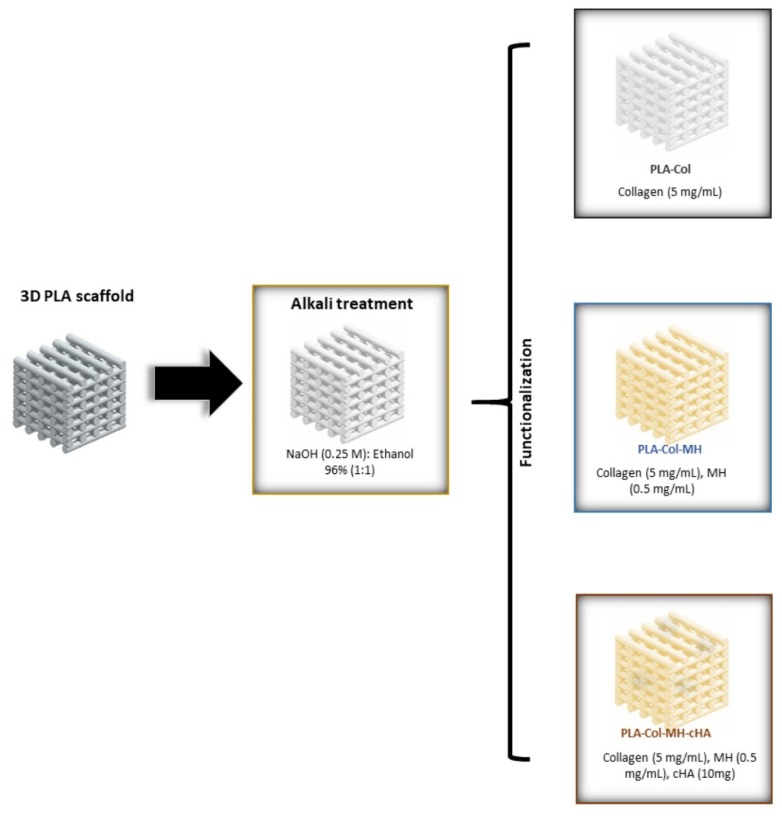
Schematic diagram of the experimental procedure for the multifunctionalization of the scaffolds after 3D-printing by using a simple coating process (PLA: polylactide; MH: minocycline hydrochloride; cHA: citrate-hydroxyapatite nanoparticles). Reprinted from [98] with permission from Elsevier.

Currently, intensive work is underway on the possibility of 3D printing composite materials. This technique involves the design of customized structures and enables effective filling of bone defects [98]. Additive manufacturing (AM) is another rapidly growing area, which includes 3D printing using computer technology (CAD). It enables the design of 3D structure composites at the micro and nano-scale, layer by layer, matching the size and number of pores, plus the shape to fit the implant to the bone defect [17,99]. For example, in [98], the 3D-printing and additive manufacturing technique was used to develop new poly-lactic scaffolds coated with Col/HA composite to give them biomimetic properties (Figure 7). Coatings were additionally enriched in an antibiotic, minocycline, to provide antibacterial protection. As a result, material with a high biocompatibility and good antibiotic release parameters was obtained. The literature reports that the best 3D printing method is the low-temperature additive manufacturing method (LTAM). The most advantageous crosslinking process for hybrid materials was taken into account, as was the possibility of introducing various bioactive molecules without destroying their structures. According to current knowledge, 3D printing seems to provide better material porosity and better control over this parameter. Comparing 3D printed materials with non-printed materials, the former was more conducive to the proliferation of bone marrow stromal cells and improved osteogenic results in vitro [100,101].

## 6. Conclusions

Creating biomimetic implant materials is one of the challenges faced by modern chemistry and material engineering. Biologically inspired HA/Col composites seem very promising materials to replace autologous bone grafts. Analyzing their chemical, physicochemical, mechanical and biological properties, we observe that by modifying the methods of obtaining composites, and the composition of hydroxyapatite, the greatest similarity to physiological bone tissue is achieved. Among other composite materials used in orthopedic surgery, HA/Col composites are distinguished by good strength and flexibility, and above all else, high biocompatibility, bioactivity, osteoconductivity and bioresorbability. A review of previous studies, as well as visible new trends (3D printing, addition of medicinal substances) and a steady increase in interest in this topic, confirm that HA/Col composites have great potential in the treatment of bone defects and diseases.
